# The distal nephron biomarkers associate with diabetic kidney disease progression

**DOI:** 10.1172/jci.insight.186836

**Published:** 2025-06-23

**Authors:** Christina L. Tamargo, Steven G. Coca, Heather Thiessen Philbrook, David G. Hu, Joachim H. Ix, Michael G. Shlipak, Linda F. Fried, Orlando M. Gutierrez, Sushrut S. Waikar, Sarah J. Schrauben, Jeffrey R. Schelling, Peter Ganz, Paul L. Kimmel, Jason H. Greenberg, Rajat Deo, Ayumi Takakura, Ramachandran S. Vasan, Joseph V. Bonventre, Chirag R. Parikh

**Affiliations:** 1 Division of Nephrology, Johns Hopkins University School of Medicine, Baltimore, Maryland, USA.; 2 Barbara T. Murphy Division of Nephrology, Icahn School of Medicine at Mount Sinai, New York, New York, USA.; 3 Division of Nephrology-Hypertension, Department of Medicine, UCSD, San Diego, California, USA.; 4 VA San Diego Healthcare System, San Diego, California, USA.; 5 Department of Medicine, UCSF, San Francisco, California, USA.; 6 San Francisco VA Health Care System, San Francisco, California, USA.; 7 Renal Section, VA Pittsburgh Healthcare System, Pittsburgh, Pennsylvania, USA.; 8 Division of Renal-Electrolyte, Department of Medicine, University of Pittsburgh School of Medicine, Pittsburgh, Pennsylvania, USA.; 9 Department of Medicine and; 10 Department of Epidemiology, University of Alabama at Birmingham, Birmingham, Alabama, USA.; 11 Section of Nephrology, Boston Medical Center and Boston University Chobanian & Avedisian School of Medicine, Boston, Massachusetts, USA.; 12 Department of Medicine, Perelman School of Medicine at the University of Pennsylvania, Philadelphia, Pennsylvania, USA.; 13 Department of Physiology and Biophysics, School of Medicine, Case Western Reserve University, Cleveland, Ohio, USA.; 14 Division of Cardiology, Zuckerberg San Francisco General Hospital, San Francisco, California, USA.; 15 National Institute of Diabetes and Digestive and Kidney Diseases, NIH, Bethesda, Maryland, USA.; 16 Section of Pediatric Nephrology, Yale University School of Medicine, New Haven, Connecticut, USA.; 17 Division of Cardiovascular Medicine, Perelman School of Medicine at the University of Pennsylvania, Philadelphia, Pennsylvania, USA.; 18 Division of Renal Medicine and Engineering in Medicine Division, Brigham and Women’s Hospital, Boston, Massachusetts, USA.; 19 Department of Medicine, Harvard Medical School, Boston, Massachusetts, USA.; 20 The University of Texas School of Public Health San Antonio, San Antonio, Texas, USA.; 21 Department of Medicine, Boston University School of Medicine, Boston, Massachusetts, USA.

**Keywords:** Clinical Research, Nephrology, Chronic kidney disease, Diabetes

## Abstract

While urinary biomarkers show promise in predicting diabetic kidney disease (DKD) progression, distal tubular markers remain understudied. We investigated the association of distal tubule markers, epidermal growth factor (EGF) and uromodulin (UMOD), with DKD progression in the Veterans Affairs Diabetes in Nephropathy (VA NEPHRON-D) clinical trial.METHODS. We used Cox regression models to evaluate the association between each biomarker and DKD progression and the relationship between change over time in biomarker and DKD progression. We used mixed models to investigate biomarker levels at baseline, 12 months, and over time and their relationships with longitudinal eGFR change.RESULTS. Participants (*n* = 1,116) had type 2 diabetes, urine albumin-to-creatinine ratio (UACR) ≥ 300 mg/g, and eGFR 30–89.9 mL/min/1.73 m^2^. Mean age was 65 years, mean eGFR was 56 (SD 19) mL/min/1.73 m^2^, and median UACR was 840 (IQR 424–1,780) mg/g. One hundred forty-four participants (13%) had DKD progression over a median follow-up of 2.2 (1.3–3.1) years. Higher baseline EGF and UMOD were independently associated with a lower risk of DKD progression (adjusted HR 0.68, 95% CI 0.47, 0.99 and 0.85, [0.75, 0.98] per 2-fold higher concentration of EGF and UMOD, respectively). Serial biomarker measurements were performed at baseline and 12 months, and a slower decline in biomarkers was associated with a lower risk of DKD progression when adjusted for baseline biomarker levels.CONCLUSION. Urinary EGF and UMOD may serve as valuable prognostic biomarkers in DKD.TRIAL REGISTRATION. ClinicalTrials.gov NCT00555217.FUNDING. NIH U01DK102730, U01DK103225, K23 DK118198, R01DK137087, U01DK103225, R37DK039773, U01DK114866, U01DK106962, U01DK129984, and R01DK093770; National Institute of Diabetes and Digestive and Kidney Diseases contract U01DK106965.

## Abstract 2

Biomarkers like creatinine can provide insight into disease states. EGF and UMOD are two urine biomarkers that we found predict progression of diabetic kidney disease.

## Graphical abstract

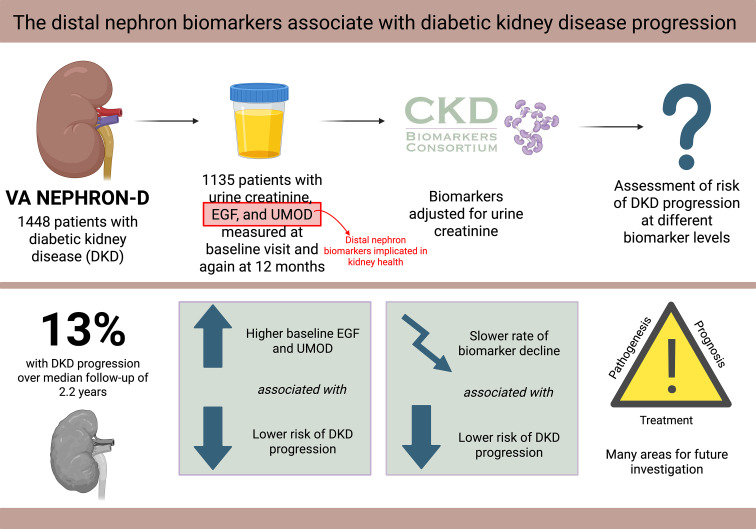

## Introduction

Diabetic kidney disease (DKD) affects 30% of individuals with type 1 diabetes and 40% of individuals with type 2 diabetes, thereby affecting hundreds of millions worldwide (1). Current evaluation for DKD diagnosis and prognosis is limited. The most definitive test for diagnosing DKD is a kidney biopsy, but it is rarely done unless the diagnosis is uncertain, as there are associated risks and complications (2–4). DKD more typically refers to a reduction in estimated glomerular filtration rate (eGFR) (i.e., eGFR < 60 mL/min/1.73 m^2^) or the presence of albuminuria (urine albumin-to-creatinine ratio [UACR] > 30 mg/g) in the setting of diabetes without another identifiable cause, but eGFR and albuminuria have diagnostic and prognostic limitations. For instance, eGFR is determined indirectly and has well-described inaccuracies (5); patients with DKD may have preserved eGFR early in their course despite extensive damage; levels of albuminuria are variable and may spontaneously regress, though even remitted albuminuria is associated with eGFR decline (6–8); and DKD can progress among persons with absent or low levels of albuminuria (9, 10). Furthermore, eGFR and albuminuria primarily reflect glomerular filtration function; however, the degree of interstitial fibrosis and tubular atrophy on kidney biopsy is one of the strongest prognostic markers for kidney failure in diabetes, and the degree of interstitial fibrosis is only partially captured by eGFR and albuminuria (11–14). Given the prevalence of DKD, the limitations of eGFR and albuminuria, and the potential for medical intervention for those at high risk of DKD development and progression (15), there is a need to identify additional biomarkers to improve our understanding of DKD progression and potentially our clinical management.

Urinary biomarkers linked to chronic kidney disease (CKD) and DKD have been identified (3, 9, 16–20). Most of these biomarkers, such as kidney injury molecule-1 and liver-type fatty acid-binding protein, are produced in the proximal tubule (21); distal tubule biomarkers have not been examined as closely. The distal tubule plays a key role in electrolyte and fluid balance, and damage to this segment of the nephron can contribute to CKD progression. Uromodulin (UMOD) and epidermal growth factor (EGF) have emerged as essential proteins of kidney health, and higher concentrations are associated with better tubule function and a lower risk of adverse kidney outcomes (22–25). As EGF and UMOD are produced mainly in the thick ascending limb and distal convoluted tubule, examining these biomarkers together can assess the health of the distal nephron (26–31). Both EGF and UMOD have been studied in diabetic and nondiabetic individuals (32), but few studies have examined their long-term prognostic value, especially in DKD. To our knowledge, only one study has done multiple measurements of EGF with longitudinal follow-up in the setting of diabetes (33), and none has done so for UMOD. Thus, we sought to assess baseline and 12-month EGF and UMOD levels in a large population of people with DKD from the Veterans Affairs Diabetes in Nephropathy (VA NEPHRON-D) study and to explore their associations with kidney disease progression.

## Results

### Participant characteristics.

Characteristics of participants with both biomarkers and all covariates available at the time of randomization are listed in Table 1. At baseline, 1,135 participants had EGF levels, and 1,127 had UMOD levels. Twelve participants were missing covariate data, leaving 1,116 participants for the complete case analysis. The mean age was 65 (SD 8) years, 99% were male, 21% identified as Black, and 9% identified as Hispanic. Most participants (63%) had stage 3 CKD (eGFR 30–59.9 mL/min/1.73 m^2^) at baseline; the mean eGFR was 56 (SD 19) mL/min/1.73 m^2^, and the median UACR was 840 (IQR 424–1780) mg/g creatinine. Treatment assignment was similar among those included, with 555 (49.7%) in the combination therapy group and 561 (50.3%) in the losartan-only group.

### Relationship between urine biomarkers, eGFR, and albuminuria at randomization.

Baseline EGF and UMOD levels were positively correlated (*r* = 0.24, *P* < 0.001) (Supplemental Figure 1; supplemental material available online with this article; https://doi.org/10.1172/jci.insight.186836DS1). Each biomarker was positively correlated with baseline eGFR (*r* = 0.39 for EGF, *r* = 0.26 for UMOD) and negatively correlated with UACR (*r* = –0.19 for EGF, *r* = –0.28 for UMOD) (all *P* < 0.001).

### Associations of baseline urine biomarkers with DKD progression.

**Figure 1:**
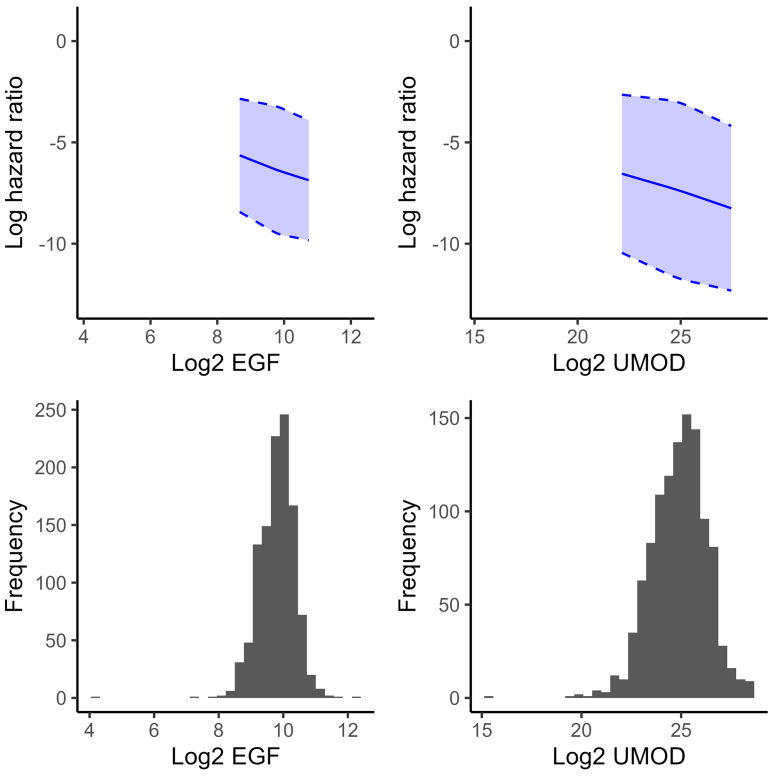
Relationship between baseline urine biomarker levels and hazard of DKD progression. Splines are truncated to the 2.5^th^ and 97.5^th^ percentiles, representing 95% of the data. Splines are unadjusted. EGF, epidermal growth factor; UMOD, uromodulin.

A total of 144 participants (13%) reached the endpoint of eGFR decline or ESKD over the median 2.2-year follow-up. In continuous models, there were significant associations between both biomarkers and DKD progression in unadjusted and fully adjusted models (Table 2). Each 2-fold higher baseline concentration of EGF or UMOD was associated with a lower risk of kidney function decline or ESKD in fully adjusted models (adjusted HR 0.68, [0.47, 0.99] for EGF and 0.85, [0.75, 0.98] for UMOD). The association between log_2_-transformed baseline EGF and UMOD levels and DKD progression was linear (Figure 1).

When incorporating baseline levels of both biomarkers into the continuous models, HRs were minimally affected, suggesting these biomarkers are fairly independent of one another. Higher UMOD remained associated with lower risk in all models. The pattern in EGF was similar and the point estimate was only modestly affected by UMOD adjustment; however, the association of EGF and DKD progression in the fully adjusted model just missed statistical significance.

### Associations of 1-year changes in urine biomarkers with DKD progression.

**Figure 2:**
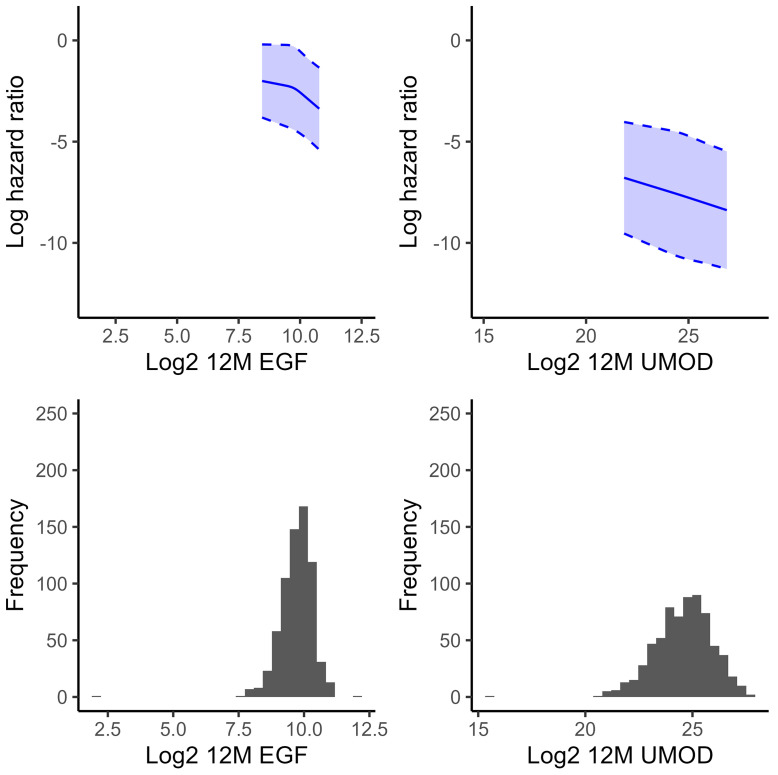
Relationship between 12-month urine biomarker levels and hazard of DKD progression. Splines are truncated to the 2.5^th^ and 97.5^th^ percentiles, representing 95% of the data. Splines are unadjusted. EGF, epidermal growth factor; UMOD, uromodulin.

EGF levels decreased by a median of –48.1 pg/mL (IQR –227, 149) from baseline to 12 months, while UMOD levels decreased by a median of –8.72 μg/mL (IQR –28.9, 8.31). When participants were divided into tertiles corresponding to worsening, stable, or improving biomarker values over time, those who had the least longitudinal decline in EGF over time, as represented by the highest tertile, had a lower risk of DKD progression (adjusted HR 0.57 [0.34, 0.94]) (Table 3 and Figure 2). While event rates were also lower for those with the least longitudinal decline in UMOD, the associations were not statistically significant. There were no significant associations for continuous models of biomarker difference for either UMOD or EGF. However, when the absolute concentrations of 12-month EGF and UMOD were adjusted for baseline EGF or UMOD, respectively, higher biomarker levels at 12 months were associated with a lower risk of DKD progression. For EGF, associations were again attenuated and no longer statistically significant after incorporating baseline UACR. Treatment assignment did not influence the proportion of participants in each tertile. Changes in eGFR and UACR over time were significantly associated with DKD progression in continuous models.

To evaluate whether treatment allocation in VA NEPHRON-D influenced associations between biomarkers and outcomes, we tested interactions of biomarker–randomized treatment arm for baseline and longitudinal analyses (Tables 2 and 3). The interaction terms were not significant for the models at baseline or at 12 months when biomarker changes were evaluated as tertiles. However, the model using 12-month continuous values to predict DKD progression was significant for UMOD but not for EGF. We have presented the hazard ratios for 12-month UMOD for each treatment arm in Supplemental Table 1.

### eGFR change over time.

Higher baseline EGF and UMOD levels were associated with a slower rate of eGFR decline (Table 4 and visualized in Supplemental Figure 2). Based on our fully adjusted mixed model, a participant with average log_2_-transformed EGF experienced an average decrease in eGFR of 11.1% (10.2%, 12.1%) per year, and every SD of EGF above the mean was associated with a decline 3.4% slower (i.e., an eGFR decline of 7.7% for EGF 1 SD above the mean). For average UMOD, the decrease in eGFR was 11.2% (10.3%, 12.2%) per year and 4.4% (3.3%, 5.5%) less for every SD above the mean. These rates of decline in eGFR were similar when EGF and UMOD at baseline were incorporated into the model. Higher EGF and UMOD levels at 12 months showed similar findings. However, longitudinal change in the biomarkers was not associated with eGFR decline in continuous models or when broken down by tertile of biomarker difference (Supplemental Table 2).

## Discussion

In this study of trial participants with type 2 diabetes, overt albuminuria, and CKD, we observed that higher baseline urine levels of EGF and UMOD, which are produced by the distal nephron and have been used as markers of distal tubular health, were each independently associated with lower risk of DKD progression. These associations were robust even after adjusting for demographic variables, clinical characteristics, eGFR, and albuminuria and were relatively unaltered even when the biomarkers were adjusted for one another. Longitudinal analyses showed that those with the least decline in EGF or UMOD over 12 months, when accounting for the baseline level of the relevant biomarker, had less subsequent eGFR decline. These findings suggest that EGF and UMOD, which characterize distal nephron tubular health, may play a role in pathways of DKD progression, above and beyond glomerular filtration and injury.

EGF is a protein isolated in many human secretory tissues and bodily fluids, such as salivary glands and tears, but is found in very low levels in human plasma (34). As it is also excreted by the kidney, specifically from cells in the thick ascending limb and distal convoluted tubule of the nephron, it can be found in large quantities in the urine (35). Lower levels of EGF are seen in tubular atrophy and interstitial fibrosis when staining kidney biopsy tissue, and due to its location of production, it is considered a marker of distal tubule function (34, 36). UMOD is the most abundant urinary protein in healthy individuals and is selectively expressed by cells mainly in the thick ascending limb of the loop of Henle, and in smaller amounts in the distal convoluted tubule, and therefore it can also be considered a specific distal tubule biomarker. As with EGF, higher levels of UMOD have been associated with a more robust tubular reserve (37). UMOD correlates closely with nephron mass, which is an additional predictor of long-term kidney function (38–40). Unlike creatinine, nephron mass is immune to the effects of hyperfiltration, so finding surrogates for nephron mass may be of diagnostic and prognostic benefit. The abundance of UMOD, its specificity for the distal kidney, and its reflection of nephron mass make it a potentially useful biomarker in DKD.

While the focus of our study was on UMOD and EGF as essential biomarkers of distal tubular health, it is also possible that one or both proteins are causal mediators of tubular and kidney health. For example, there are mutations in UMOD that result in diminished UMOD excretion that are associated with familial forms of CKD, and genome-wide association studies have demonstrated associations between the UMOD locus with the risk of developing CKD in the general population (41–44). Animal models have shown that mutations in EGF causing EGF deficiency were associated with increased albuminuria and, in a subset, severe and progressive kidney disease (45). While these causal inference analyses have not been done on a large scale in human DKD cohorts, they do support the concept that EGF and UMOD could have diagnostic and prognostic implications in CKD.

While many studies have examined correlations between these biomarkers and traditional markers of kidney function like eGFR and albuminuria, fewer have assessed the relationship of urine biomarker levels with subsequent changes in kidney function, and none to our knowledge has previously assessed longitudinal changes in these biomarkers with subsequent kidney disease risk. Most prior studies reported findings that aligned with our findings — that higher baseline levels of EGF and UMOD were associated with a lower risk of CKD progression. Only 2 studies, both on UMOD, showed the opposite: One was in a cohort of patients with type 1 diabetes without CKD (46), and the other was another cohort without baseline CKD (47). Thus, based on the totality of the evidence, EGF and UMOD, and by virtue the distal tubule, may provide unique insights with prognostic implications for DKD biology.

Most studies on urine biomarkers thus far have focused on injury biomarkers that predominantly originate from the proximal tubule. The proximal tubule is of particular interest because of its absorptive capacity and high energetic activity. It also has unique functions in DKD, as it is exposed to and reabsorbs high levels of glucose, albumin, and fatty acids; proximal tubular reabsorption of glucose and sodium in the diabetic kidney leads to suppressed tubuloglomerular feedback and thus renal vasodilation, contributing to the glomerular hyperfiltration in early DKD, which is just one of many roles of the proximal tubule in this disease state (48). This study suggests that the distal tubule could also play a role in DKD. Because the goal of kidney biopsies is to obtain the cortex, which contains predominantly proximal tubules and glomeruli, the medullary thick ascending limbs are not typically as well visualized. Distal tubule–specific urine biomarkers might therefore provide pathophysiologic information that is not otherwise accessible.

The study has many strengths but also important limitations. Over 99% of participants were male, reflecting its recruitment from US VA health systems, which limits the generalizability of the results. Because the study was stopped early, follow-up was relatively short at 2.2 years. Longer follow-up would have provided additional clinical events and potentially strengthened associations, enhancing the clinical relevance of EGF and UMOD from a prognostic perspective. As expected (49), mean eGFR was lower and median UACR was higher in tertiles with worse outcomes; there were other statistically significant differences between tertile groups at baseline in BMI, blood pressure, and hemoglobin A1c; and confounding is still possible despite adjustment as is the case in epidemiological studies. Participants all had hemoglobin A1c at or below 10.5% at baseline, so our findings may not apply to people with poorly controlled diabetes. In addition, the participants were classified as having DKD because they had diabetes, albuminuria, kidney disease, and no other known cause of their kidney disease. In the absence of kidney biopsies, however, non-DKD cannot be excluded in all cases. Nevertheless, the DKD diagnosis in this study reflects clinical practice. Due to differences in assays and case mix, we were unable to compare biomarker distributions across other CKD cohorts, such as CRIC, ARIC, Regard, and MESA (50–52). Finally, the VA NEPHRON-D trial was conducted before the discovery of sodium glucose co-transporter 2 (SGLT2) inhibitors, which have known effects on tubular health and expression of EGF (33). As SGLT2 inhibitors are now a key component of DKD care, this makes the study less generalizable in the current era but also presents opportunities for further research on the relationship between this therapy, distal tubule biomarkers, and kidney outcomes.

In summary, higher baseline urine levels of EGF and UMOD were associated with a lower risk of eGFR decline and development of ESKD in participants with DKD. These findings highlight the importance of the distal tubule in DKD prognosis and support investigation into its role in promoting kidney disease progression. Further exploration of EGF and UMOD and other aspects of distal tubular health in DKD and other CKD populations could bring new insights into DKD pathobiology with implications for therapeutic development.

## Methods

### Sex as a biological variable.

Our study examined predominantly men because it occurred in VA medical centers; however, 1% of participants were women.

### Study population.

VA NEPHRON-D was a multicenter, prospective, randomized, parallel-group trial examining the efficacy of combination therapy with ACEi and ARB versus standard treatment with ARB alone on CKD progression in participants with type 2 diabetes, UACR ≥ 300 mg/g, and eGFR 30.0 to 89.9 mL/min/1.73 m^2^, as previously described (53). Major exclusion criteria included ACEi or ARB intolerance, serum potassium > 5.5 mEq/L, suspected non-DKD, hemoglobin A1c > 10.5%, and end-stage comorbid disease (54). Between July 2008 and September 2012, 1,448 participants from VA medical centers across the United States were randomized into combination therapy (ACEi and ARB, lisinopril and losartan) versus single-therapy (losartan only) groups. The study was planned to have a 4.25-year enrollment period and 6.25 years of follow-up for all enrollees. However, the trial was stopped early due to safety concerns, mainly acute kidney injury and hyperkalemia, with a median follow-up of 2.2 (IQR 1.3–3.1) years.

VA NEPHRON-D participants were included in our analyses if urine creatinine, EGF, and UMOD were successfully measured at their baseline visit. Of the 1,448 participants included in VA NEPHRON-D, 1,135 (78.4%) met these criteria. We also measured EGF and UMOD at the 12-month visit in 674 participants; these measures were used to calculate the changes in each protein over the first year of the trial.

### Sample collection and biomarker measurement.

We collected urine samples at baseline (the time of randomization) and stored them at –80°C until biomarker measurement. Measurements were based on CKD Biomarkers Consortium protocols (55, 56). UMOD was measured via the Meso Scale Discovery platform (Meso Scale Diagnostics, LLC [MSD]), which uses electrochemiluminescence detection combined with patterned assays. Baseline and 12-month UMOD were measured in diluted samples in duplicate on the MSD R-PLEX, and the mean value was used, with assay range 240–4,000,000 pg/mL, and intra- and interassay coefficients of variation 4.8% and 5.3%, respectively. All values of UMOD and EGF were within the limits of detection. Personnel performing biomarker measurements were masked to participant data. Baseline and 12-month EGF measurements were completed in duplicate using a Luminex multiplex assay (Luminex Corporation) on a Bio-Rad BioPlex 200 system with appropriate dilutions, and the mean value was also used. The assay detection range was 0.98–4,000 pg/mL, and intra- and interassay coefficients of variation were <10%. Samples were diluted with a commercially available (MSD) Diluent 37 buffer for UMOD measurements and diluted 1 to 4 with buffer containing 100 mM of NaCl and HEPES (pH 7.4) to maintain pH for the EGF Luminex assay. There was no interference due to glucose (up to 500 mg/dL), hemoglobin (40 mg/dL), albumin (200 mg/dL), or bilirubin (10 mg/ dL). Biomarker concentrations were adjusted for urine creatinine concentration (i.e., the ratio of the urine biomarker to urine creatinine), which was measured by the Jaffe reaction using a Randox Imola instrument with a coefficient of variation of <4% and aligned to their baseline measurements to assess long-term changes. These assays were run at the Brigham and Women’s Hospital Renal BioCon Central Laboratory in Boston, Massachusetts.

### Other study variables.

The covariates included age, sex, race, ethnicity, coronary artery disease, congestive heart failure, diabetic retinopathy, BMI, systolic and diastolic blood pressure, lipids (total cholesterol, LDL-C, HDL-C, and triglycerides), hemoglobin A1c, eGFR, albuminuria, and antihypertensive therapy, all at baseline, and randomized treatment assignment.

Age, sex, race, and ethnicity were self-reported. BMI was calculated as kilograms divided by the square of meters; height and weight were recorded locally. Blood pressure was measured twice at least 1 minute apart with participants seated after at least 5 minutes of rest. The average of the 2 readings was used. If there was a >5 mmHg difference in systolic or diastolic blood pressure between these 2 readings, a third measurement was obtained and the median was used. Lipids, hemoglobin A1c, and UACR were measured locally at the participating VA centers. Serum creatinine was measured every 3 months by the Siemens Dimension RxL chemistry system at a central laboratory (University of Maryland).

### Study outcomes.

The outcome was an eGFR decline of ≥30 mL/min/1.73 m^2^ if eGFR at randomization was ≥60 mL/min/1.73 m^2^, eGFR decline of ≥50% if eGFR at randomization was <60 mL/min/1.73 m^2^, or ESKD (defined as the need for maintenance dialysis or kidney transplant, or eGFR < 15 mL/min/1.73 m^2^), which were the secondary renal outcomes in the original VA NEPHRON-D trial. Given the study population, this outcome was used as a surrogate for DKD progression. eGFR was calculated using the Modification of Diet in Renal Disease Study equation (57).

### Statistics.

We described the study population using mean (SD) for normally distributed continuous variables, median (IQR) for skewed continuous variables, and frequency (proportion) for categorical variables. We assessed the correlation between the 2 urinary biomarkers (not indexed, log_2_-transformed) at baseline, as well as with eGFR and UACR, using Pearson’s correlation coefficients. We calculated mean event rates per 1,000 person-years for the overall cohort.

Cox regression models were used to evaluate the association between each biomarker and DKD progression. Model 1 adjusted for urine creatinine, demographic variables (age, sex, race, ethnicity), clinical characteristics (BMI, systolic blood pressure, hemoglobin A1c), and treatment assignment at baseline. Model 2 was further adjusted for UACR and eGFR as current clinical measures of kidney function. We also fit a model that included both UMOD and EGF at baseline. We performed a complete case analysis and excluded participants for whom ethnicity (*n* = 1), BMI (*n* = 1), or hemoglobin A1c (*n* = 10) were unavailable.

For participants with biomarker measurements available at both baseline and 12 months, we used the change in each biomarker as an additional exposure and applied the same models. After log_2_-transforming biomarkers, we categorized participants into tertiles based on the difference between 12-month and baseline measurements. For these analyses, time-to-event was adjusted such that the 12-month visit date was considered time 0, and participants who experienced the event before 12 months were removed; this left 100 events after 12 months in the EGF cohort and 104 in the UMOD cohort. We also tested interactions by randomized treatment arm for all analyses, including at baseline, at 12 months, and per change over time. We used model 2 and added an interaction term for treatment for these analyses.

We did additional mixed models examining associations of baseline biomarker level, 12-month biomarker level, and change in biomarker level over time with longitudinal eGFR change. EGF and UMOD were again log_2_-transformed and subsequently standardized, and we calculated the percentage change in eGFR per year at average values of these biomarkers as well as at incremental SDs from the mean. Participants who developed ESKD (*n* = 56) were assigned an eGFR of 10 at their mortality date.

Analyses were conducted using SAS software, version 9.4 (SAS Institute) and R version 4.2.1 (R Foundation for Statistical Computing). *P* < 0.05 was considered statistically significant.

### Study approval.

All participants provided written informed consent before inclusion, and the human rights committee at the West Haven VA Cooperative Studies Program Coordinating Center (West Haven, Connecticut, USA) and institutional review boards at each participating site approved the study.

### Data availability.

Summary statistics for Figures 1 and 2 and Supplemental Figure 1 are reported in the Supporting Data Values file. The data supporting the findings of this study are from the Department of Veterans Affairs Cooperative Studies Program, which were used under agreement for the current study and are not publicly available. Data may be requested from the Department of Veterans Affairs Cooperative Studies Program.

## Author contributions

CLT performed conceptualization, methodology, formal analysis, writing (original draft, review, editing), and visualization. HTP developed methodology and software and performed validation, formal analysis, data curation, writing (review, editing), visualization, and supervision. DGH developed methodology and software and performed validation, formal analysis, data curation, writing (review, editing), visualization. AT performed investigation and writing (review, editing). JVB contributed to investigation, resources, and writing (review, editing). CRP contributed to conceptualization, methodology, resources, writing (review, editing), supervision, and funding acquisition. SGC, JHI, MGS, LFF, OMG, SSW, SJS, JRS, PG, PLK, JHG, RD, and VSR contributed to writing (review, editing).

## Supplementary Material

Supplemental data

ICMJE disclosure forms

Supporting data values

## Figures and Tables

**Table 1:**
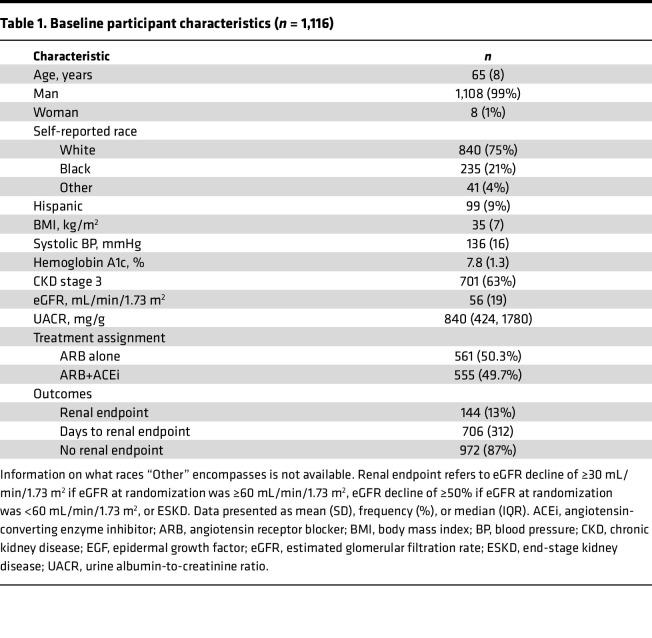
Baseline participant characteristics (*n* = 1,116)

**Table 2:**
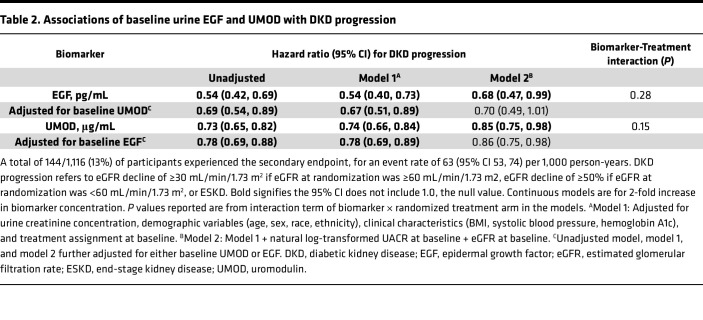
Associations of baseline urine EGF and UMOD with DKD progression

**Table 3:**
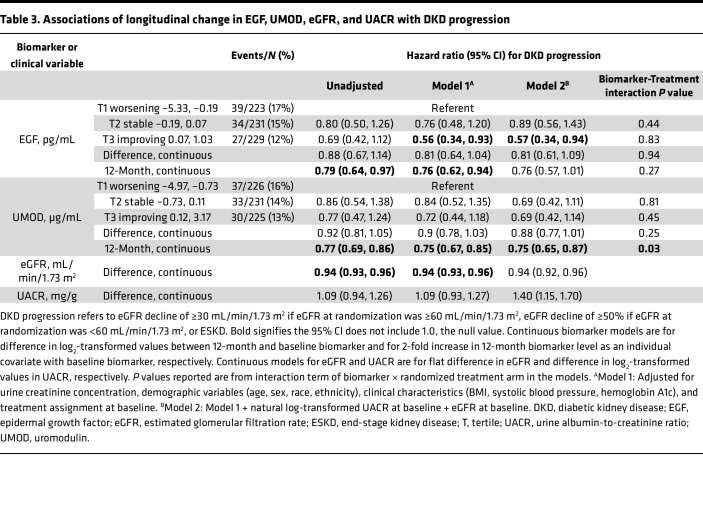
Associations of longitudinal change in EGF, UMOD, eGFR, and UACR with DKD progression

**Table 4:**
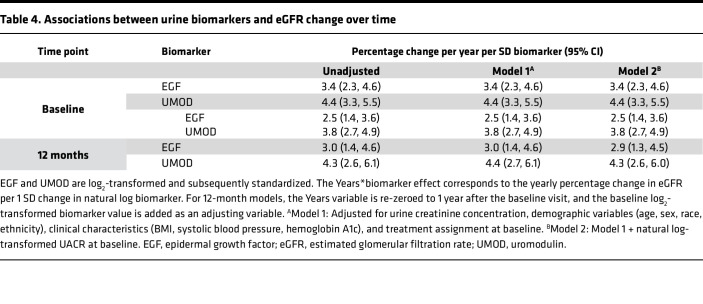
Associations between urine biomarkers and eGFR change over time

## Version 1 06/23/2025

Electronic publication
