# Supplementary material for: The distal nephron biomarkers associate with diabetic kidney disease progression
Source: JCI Insight. 2025 Jun 23;10(12):e186836. doi: 10.1172/jci.insight.186836 (PMC12220953; doi:10.1172/jci.insight.186836)
Supplement: Supplemental data [file jciinsight-10-186836-s114.pdf]

SUPPLEMENTAL MATERIAL

TITLE: The distal nephron biomarkers associate with diabetic kidney disease progression

AUTHORS: Christina L Tamargo[1], Steven G Coca, DO[2], Heather Thiessen Philbrook[1], David G Hu[1], Joachim H Ix[3,4], Michael G Shlipak[5,6], Linda F Fried[7,8], Orlando M Gutierrez[9,10], Sushrut S Waikar[11], Sarah J Schrauben[12], Jeffrey R Schelling[13], Peter Ganz[5,14], Paul L Kimmel[15], Jason H Greenberg[16], Rajat Deo[17], Ayumi Takakura[18,19], Vasan S Ramachandran[20,21], Joseph V Bonventre[18,19], Chirag R Parikh[1]

Table of Contents

Conflicts of interest / author disclosure information

Supplementary Table S1. [Association of 12M UMOD with DKD progression by treatment group.]

Supplementary Table S2. [Associations between longitudinal urine biomarker change and eGFR change over time.]

Supplementary Figure S1. [Correlations between urine biomarkers, eGFR, and albuminuria at randomization.]

Supplementary Figure S2. [eGFR trajectories by baseline urine biomarker level.]

SGC reports consulting fees from Renalytix, Takeda, Nuwellis, Vifor, Bayer, Boehringer-Ingelheim, Reprieve Cardiovascular, Axon, and 3ive; ownership interest in Renalytix and pulseData; research funding from Renalytix, ProKidney, Renal Research Institute, and XORTX; patents/royalties from Renalytix (filed patents PCT/US2021/018030, Urinary Plasminogen as a Marker of Disease Progression in Human Glomerular Disease, and US Patent Application No. 63/510,558); advisory or leadership roles in Renalytix; and other interests or relationships as associate editor for Kidney360 and on editorial boards of JASN, CJASN, and Kidney International. SGC is adjudicator for the Critical Path Institute's Predictive Safety Testing Consortium Nephrotoxicity Working Group. JHI received consulting fees from AstraZeneca, Bayer, Jnana (Otsuka), and Marinus; investigator-initiated research grant support from the Juvenile Diabetes Research Foundation; and travel support from Kidney Disease: Improving Global Outcomes. MGS reports honoraria from AstraZeneca, Bayer, and Boehringer Ingelheim and research support from Bayer.

LFF has received grants from AstraZeneca, was previously a data safety monitoring board member for Novo Nordisk and CSL Behring, and has had leadership roles in the American Society of Nephrology. OMG reports honoraria from Ardelyx, Amgen, and AstraZeneca. SJS is on the editorial board of AJKD. JRS has a leadership role in the Kidney Foundation of Ohio. PG serves on the medical advisory board to SomaLogic, for which he receives no financial remuneration. PLK received royalties as the co-editor of *Chronic Renal Disease* and *Psychosocial Aspects of Chronic Kidney Disease* and an advance from Mayo Clinic Press for *The Body's Keepers*. JVB is a consultant to Sarepta, Praxis, Nimbus, and GentiBio and owns equity in Innoviva, MediBeacon, DxNow, Verinano, Autonomous Medical Devices, and Renalytix. CRP is an advisory member of Alexion, Bayer, and Otsuka.

Supplementary Table S1. Association of 12-month UMOD level with DKD progression by treatment group.

Model	Treatment	Hazard Ratio (95% CI)
**Unadjusted**	ACEI	**0.618 (0.506, 0.756)**
	ACEI+ARB	0.863 (0.724, 1.029)
**Model 1**	ACEI	**0.604 (0.490, 0.745)**
	ACEI+ARB	0.842 (0.706, 1.004)
**Model 2**	ACEI	**0.625 (0.499, 0.783)**
	ACEI+ARB	0.865 (0.694, 1.078)

ACEI, angiotensin-converting enzyme inhibitor; ARB, angiotensin receptor blocker; CI, confidence interval; DKD, diabetic kidney disease; UMOD, uromodulin. Bold signifies the 95% CI does not include 1.0, the null value.

Supplementary Table S2. Associations between longitudinal urine biomarker change and eGFR change over time.

Biomarker	Effect	Percent Change (95% CI)		
		Unadjusted	Model 1[A]	Model 2[B]
EGF Difference	Years	-9.6 (-11.0, -8.1)	-9.5 (-11.0, -8.1)	-9.5 (-10.9, -8.0)
	Years*EGF Difference	1.3 (-2.5, 5.2)	1.3 (-2.5, 5.2)	1.2 (-2.5, 5.1)
EGF Difference, Tertiles	Years	-11.4 (-13.9, -8.8)	-11.3 (-13.8, -8.8)	-11.3 (-13.8, -8.7)
	Years*EGF Difference T1		referent	
	Years*EGF Difference T2	3.4 (-0.7, 7.5)	3.3 (-0.7, 7.4)	3.4 (-0.6, 7.6)
	Years*EGF Difference T3	2.3 (-1.7, 6.6)	2.3 (-1.7, 6.6)	2.3 (-1.8, 6.6)
UMOD Difference	Years	-9.6 (-11.2, -8.1)	-9.6 (-11.1, -8.1)	-9.6 (-11.1, -8)
	Years*UMOD Difference	0.1 (-1, 1.2)	0.1 (-1, 1.2)	0 (-1.1, 1.1)
UMOD Difference, Tertiles	Years	-9.9 (-12.4, -7.3)	-9.9 (-12.4, -7.3)	-9.7 (-12.2, -7.1)
	Years*UMOD Difference T1		referent	

Years*UMOD Difference T2	-0.1 (-4, 4)	-0.1 (-4.1, 3.9)	-0.4 (-4.3, 3.7)
Years*UMOD Difference T3	0.8 (-3.2, 4.9)	0.8 (-3.1, 4.9)	0.7 (-3.2, 4.9)

EGF, epidermal growth factor; eGFR, estimated glomerular filtration rate; T, tertile; UMOD, uromodulin. [A]Model 1: Adjusted for urine creatinine concentration, demographic variables (age, sex, race, ethnicity) and clinical characteristics (BMI, systolic blood pressure, hemoglobin A1c). [B]Model 2: Model 1 + natural log-transformed UACR at baseline.
EGF and UMOD are log2-transformed and subsequently standardized. The Years effect corresponds to the percent change in eGFR per year at average values of log2 biomarker change from baseline to 12 months, and the Years*biomarker difference effect corresponds to the yearly percent change in eGFR per one standard deviation change in longitudinal log2 biomarker change. The Years variable is re-zeroed to 1 year after the baseline visit.

Supplementary Figure S1. Correlations between urine biomarkers, eGFR, and albuminuria at randomization.

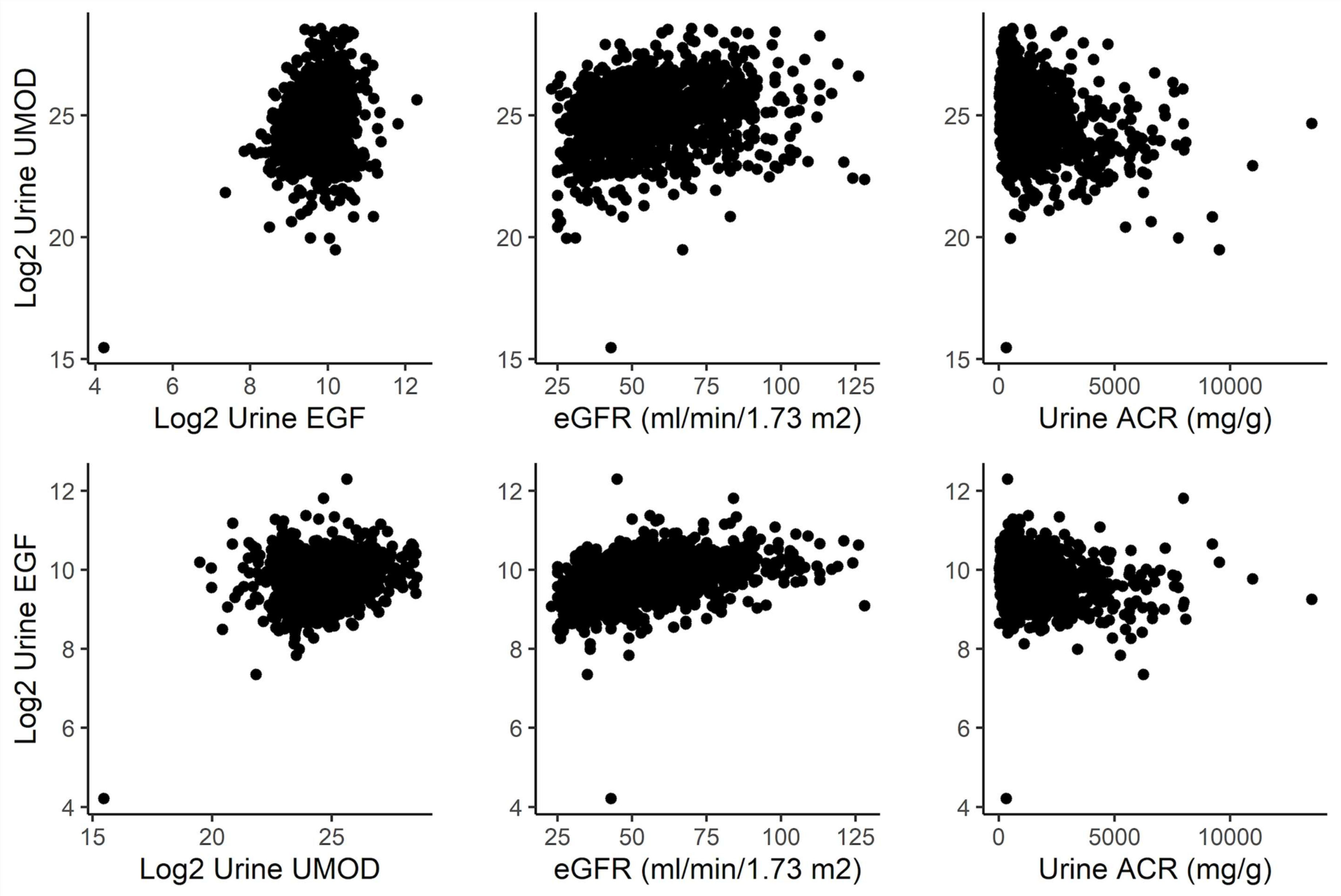

ACR, albumin-to-creatinine ratio; EGF, epidermal growth factor; eGFR, estimated glomerular filtration rate; UMOD, uromodulin.

Supplementary Figure S2. eGFR trajectories by baseline urine biomarker level.

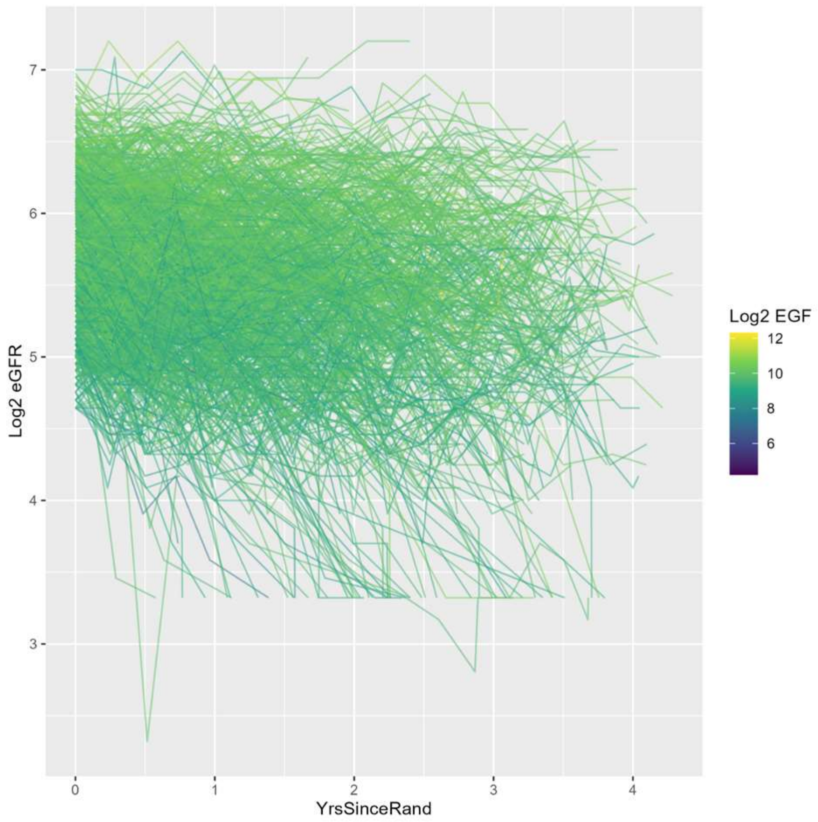

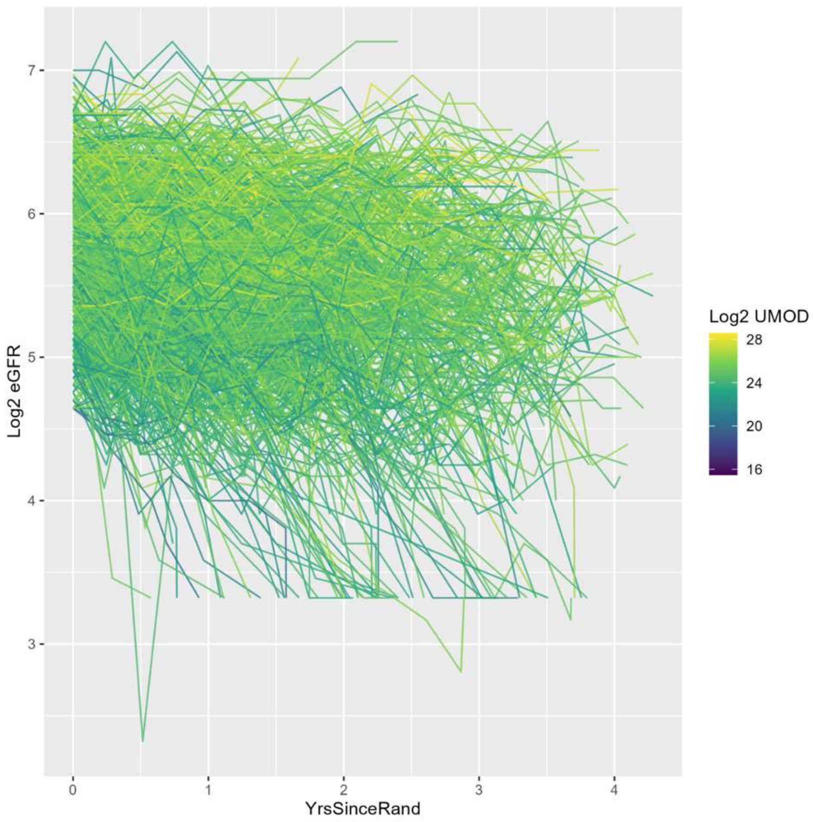

EGF, epidermal growth factor; eGFR, estimated glomerular filtration rate; UMOD, uromodulin. Spaghetti plots are color coordinated by baseline biomarker level.
